# Analysis of the Diet Quality and Nutritional State of Children, Youth, and Young Adults with Prader–Willi Syndrome: A Polish Multiple Case Study

**DOI:** 10.3390/nu15173811

**Published:** 2023-08-31

**Authors:** Michał Skrzypek, Katarzyna Kowal, Paweł Glibowski, Grzegorz Dzida, Karolina Nowosad, Renata Krzyszycha, Klaudia Soczyńska, Olga Przybysz

**Affiliations:** 1 Faculty of Health Sciences, Vincent Pol University in Lublin, Choiny 2 Str., 20-816 Lublin, Poland; 2 Collegium Medicum, Jan Długosz University in Czestochowa, 13/15 Armii Krajowej Str., 42-200 Częstochowa, Poland; k.kowal@ujd.edu.pl; 3 Department of Biotechnology, Microbiology and Human Nutrition, University of Life Sciences in Lublin, 8 Skromna Str., 20-704 Lublin, Poland; pawel.glibowski@up.lublin.pl (P.G.); karolina.nowosad@up.lublin.pl (K.N.); 4 Diabetes Division, Chair and Department of Internal Diseases, Medical University of Lublin, 16 Staszica Str., 20-081 Lublin, Poland; grzegorz.dzida@wp.pl; 5 Department of Clinical Dietetics, Medical University of Lublin, 1 Al. Racławickie Str., 20-059 Lublin, Poland; renata.krzyszycha@umlub.pl; 6 Nutrition Division, Independent Public Clinical Hospital No 4, 8 Jaczewskiego Str., 20-954 Lublin, Poland; klarysa1196@gmail.com (K.S.); olgaprzybysz2506@gmail.com (O.P.)

**Keywords:** Prader–Willi syndrome, diet quality, nutritional status, dietary intake

## Abstract

Given the lack of data on dietary quality in young individuals with Prader–Willi syndrome (PWS) in Poland, a multiple case study was conducted in which anthropometric measurements and 7-day dietary records were collected from 20 subjects with PWS. The study group consisted of 8 females and 12 males with a mean age of 14.8 years and a mean BMI of 21.6. Based on BMI analysis, five subjects were overweight, including two subjects who were obese. The study showed that 35% of the subjects had energy intakes above the recommended levels. Protein deficiency was found in one subject in the analyzed diets. However, fat intake was excessive in four subjects, and the majority exceeded the recommended intake of saturated fatty acids. Vitamin E and B_12_ deficiencies were found in 40% and 85% of the subjects, respectively. All subjects had inadequate intakes of vitamin D and iodine, while the majority had deficiencies in sodium and copper intakes. Calcium intake was deficient in 35% of the subjects. However, most subjects met recommendations for the intakes of other minerals, vitamins, and fiber. These findings confirm the suboptimal dietary patterns of Polish individuals with PWS, with deficits observed in the intake of certain vitamins and minerals.

## 1. Introduction

Prader–Willi syndrome (PWS) is a rare genetic disorder with an estimated incidence of 1:10,000–1:25,000 live births. PWS is the most common genetic cause of life-threatening obesity. It is estimated that there are approximately 350,000–400,000 people worldwide living with PWS [1]. Around 70% of PWS cases are attributed to a paternally transmitted deletion of chromosome 15q11-q13 (genetic type I), while approximately 25% of cases are characterized by a maternal uniparental disomy of chromosome 15 (mUPD) (genetic type II). Other cases of PWS result from genetic abnormalities in the 15q11-q13 region, such as imprinting defects or paternal chromosome 15 translocations [1,2]. The underlying genetic cause of PWS is the absence of active paternal genes located on chromosome 15 in the 15q11-q13 region [2].

Phenotypic differences exist between genetic type I and type II of Prader–Willi syndrome (PWS). In genetic type I, the characteristic behavioral features of PWS, such as hyperphagia and mental retardation, are more pronounced. On the other hand, type II is associated with better cognitive functioning and milder behavioral problems, but it is characterized by visual acuity and stereoscopic vision disorders not observed in type I [1]. It is worth noting that within patients with a deletion (genetic type I), subgroups distinguished by deletion size (type I deletions and type II deletions) do not differ in terms of the behavioral phenotype [3].

The behavioral phenotype of Prader–Willi syndrome (PWS) is primarily characterized by significant issues related to nutrition. A prominent feature is a voracious appetite, which, when combined with a gag reflex disorder and the absence of caregiver intervention, leads to severe and treatment-resistant obesity [4]. Excessive eating can even result in life-threatening stomach rupture [5]. There are other factors that contribute to the development of obesity in individuals with PWS. These include altered body composition, with reduced body fat content due to growth hormone (GH) deficiency, as well as reduced physical activity and co-occurring mental disorders that further exacerbate eating behavior disorders. In a cohort study conducted by the Pfizer International Growth Study (KIGS) with 424 children with PWS, GH deficiency was found in 74% of the subjects. This deficiency is responsible for short stature and metabolic disorders in PWS patients, leading to a decrease in lean body mass and an increase in fat mass [6]. Children with PWS also exhibit diminished GH responses in stimulation tests and a substantial reduction in spontaneous GH secretion, ranging from 58% to 100% [7]. The main factors contributing to the reduced energy demand in PWS and the development of obesity are body composition disorders, specifically a deficiency of lean mass, and low levels of physical activity [8].

In individuals with PWS, there is a disproportionate body composition characterized by an excess of fat mass relative to lean mass. This disproportion exists regardless of body weight and significantly affects the energy requirements of individuals with PWS. As a result, every person with PWS, regardless of their body weight, exhibits obesity-like characteristics [9]. These observations led to the introduction of recombinant human growth hormone (GH) as part of the treatment for PWS in 2000, following the decision of the Food and Drug Administration (FDA). GH acts as an anabolic factor and helps correct body composition disorders. It improves the growth process, increases bone strength, enhances the proportion of lean mass in the body, reduces fat mass, improves lipid profiles, enhances cognition, and improves overall quality of life [10,11]. A meta-analysis conducted by Rosenberg et al. on the effects of GH treatment in PWS indicates that it leads to an increase in lean mass by 1.95 kg after 12 months of treatment, with a simultaneous decrease in fat mass by 2.23%, all without changes in BMI [12]. The effects of GH therapy are most pronounced in the first year of treatment [11]. The observed improvement in body composition among adults with PWS not only contributes to a reduction in their cardiovascular risk profile but also positively affects their energy requirements.

Body composition disorders in individuals with PWS contribute to reduced energy expenditure. GH treatment can modify this by increasing energy expenditure from 50% to 75% of the standard energy requirement for healthy individuals [13]. It is important to note that even with appropriate body weight for height, individuals with PWS still have abnormally high fat mass content [14]. There is some variation in the literature regarding the extent of GH’s impact on energy demand in PWS. Research conducted by Bakker et al. suggests that GH treatment only results in a slight increase in energy intake [15]. However, it is associated with beneficial improvements in body composition, as demonstrated in various research studies [15,16]. In this study, we follow the recommendations of Polish pediatric endocrinology, which proposes that GH treatment increases the energy requirement for individuals with PWS to 75% of the standard caloric requirement. This represents an increase from the 50% caloric requirement calculated based on the standard for individuals with PWS who are not undergoing GH treatment [13].

The typical phenotypic picture of PWS appears between 18 and 36 months of age. It is preceded by a phenotypically different phase of difficulty in feeding, with central hypotonia, impaired sucking reflex, and poor weight gain (“failure to thrive”, FTT) [4]. The complete manifestation of PWS primarily involves eating behavior disorders characterized by hyperphagia, which is accompanied by early-onset obesity typically occurring in the second year of a child’s life (stage 2). Additionally, individuals with PWS may experience psychomotor retardation and a low level of physical activity [1,4]. Miller et al. proposed a comprehensive, two-stage approach to delineate the behavioral and nutritional phenotype of PWS [4]. In the natural course of PWS, they identified five distinct nutritional phases. Phase 0 is characterized by intrauterine development disorders. Phase 1 occurs when the infant is flaccid, not yet obese, and experiences feeding difficulties. Phase 2 marks the onset of weight gain. Phase 3 is characterized by hyperphagia, food-seeking behavior, and a lack of satiety. Finally, phase 4, which may affect some individuals with PWS, is characterized by a sense of satiety [4].

Eating-related behavioral problems are a prominent and lifelong feature of the behavioral phenotype in individuals with PWS, necessitating food-related supervision [17]. Most patients experience temper tantrums and “rages” due to their inability to satisfy hunger. The daily lives of families revolve around the constant struggle to effectively control the eating habits of affected individuals. In cases where these efforts prove ineffective, the body weight of affected children in their late teens can reach up to 113–136 kg [5]. Ongoing pharmacological studies aim to address satiety disorders through the use of various treatments, such as ghrelin, diazoxide, oxytocin/carbetocin, glucagon-like peptide 1 agonists, serotonin–norepinephrine–dopamine reuptake inhibitors, and others [10].

To date, there are no Polish studies available that assess the quality of the diet, including nutritional intake, in children, adolescents, and young adults with PWS. The Section 4 of this study analyzes a limited number of existing studies from the international literature on this topic, as no Polish studies of this nature have been conducted.

The objective of this study was to evaluate the nutritional status and dietary component intake in the daily food consumption of children, adolescents, and young adults with PWS.

## 2. Materials and Methods

### 2.1. Sample and Procedure

Data were collected from a sample of 20 community-dwelling individuals with PWS, comprising 8 females and 12 males, with a mean age of 14.8 years (minimum 2 years 8 months, maximum 28 years, median 14, standard deviation 7.3). These individuals were recruited to participate in this research project as part of a larger sociological study investigating the experiences and quality of life of caregivers of children, adolescents, and young adults with PWS [18]. Each study participant received an individual assessment of their nutritional status and diet quality, along with dietary advice tailored to address any identified irregularities. Additionally, a 7-day diet plan was provided. A total of 20 parents/guardians of children with PWS were invited to participate in the study, and all 20 agreed, resulting in their children being included in the research. Participation in the study was voluntary and anonymous. The research sample was purposefully selected, with the exclusion criterion being the presence of significant nutritional status impairments or diseases requiring special diets.

The study protocol was approved by the Bioethics Committee at the Medical University of Lublin (Resolution No. KE-0254/19/2021, dated 28 February 2021). Informed consent was obtained from the parents/guardians of individuals with PWS. The research was conducted between 1 May 2021, and 1 September 2021.

### 2.2. Measures

The nutritional status of the subjects included in the study was assessed using anthropometric measurements, which included body weight and height. Body Mass Index (BMI) was calculated as the ratio of body weight to the square of height (in kg/m^2^). Anthropometric measurements were taken with weight measured to the nearest 0.1 kg and height to the nearest 0.1 cm. The parents/guardians of children with PWS conducted the anthropometric assessment. They received training from a medical professional on the methodology of anthropometric measurements in accordance with WHO standards [19].

To assess the nutritional status of the PWS group treated with GH, the calculated BMI value was compared to the current Polish standard, represented by a percentile grid specifically developed for children aged 3–18 of both sexes. This standard was created as part of the OLA NR13 000206 and OLAF programs PL0080, coordinated by researchers from the Children’s Memorial Health Institute in Warsaw. The programs were conducted between 2007 and 2012, based on a representative sample of Polish children aged 3–18 [20]. Cut-off points for overweight and obesity were adopted according to the International Obesity Task Force (IOTF) criteria. The nutritional status of children at the age of 2 years and 8 months was assessed using the WHO standard dedicated to the assessment of physical development in children aged 0–5 years, specifically the girls’ BMI percentile chart. This standard was developed based on data from The WHO Multicentre Growth Reference Study conducted between 1997 and 2003 [21]. For adults (*n* = 7), the nutritional status was assessed using the WHO classification based on BMI categories: below 18.5—underweight; 18.5–24.9—normal weight; 25.0–29.9—preobesity; 30.0–34.9—obesity class I; 35.0–39.9—obesity class II; above 40—obesity class III [22]. This group comprised three adults who were not treated with GH, including one 25-year-old male, one 21-year-old female, and one 28-year-old male.

To assess the quality of the subjects’ diet, a 7-day dietary record was used. The record was completed by the parents/guardians of the subjects with PWS for 5 working/school days and 2 weekend/free days. The caregivers of the PWS subjects selected specific days for recording the diet, aiming to capture typical eating patterns. During the initial meeting with the research team, detailed instructions on completing the current quotations questionnaire were provided to the parents/guardians. To determine portion sizes of consumed foods and meals, an album of photographs featuring products and dishes commonly used in Poland was utilized [23]. The parents/guardians of the subjects were then requested to send the completed questionnaire via email. Certified dieticians (K.N., R.K., K.S., O.P.) reviewed each completed questionnaire for accuracy. Days with incomplete records were excluded from the analysis.

The nutritional value of consumed products and dishes was calculated using the commercial computer program Kcalmar.pro (Hermax, Poland), based on the 7-day dietary records provided by the subjects’ caregivers. The dietary records (in Polish) are available upon request. The nutritional data used in the Kcalmar.pro program are sourced from the Polish database of the nutritional values of food products and dishes [24]. The energy value of daily food rations (DFRs) and nutrient intake levels in the diets of the subjects with PWS were calculated and compared to the nutritional standards for the Polish population, considering age-specific standards. The obtained results were also compared to national consumption standards based on the estimated average requirement (EAR) for specific dietary components (when available) using age-appropriate standards [25]. If the EAR level was not specified, the adequate intake (AI) level was used as a reference.

In assessing nutrient intake, we utilized a conservative approach, which involved analyzing the difference between the observed intake and the EAR level for age and gender [26]. We adopted an arbitrary criterion that the allowable deviation from the norm should not exceed 10%. If an individual’s intake fell below 90% of the EAR standard, it was considered deficient. For AI, a different interpretation was applied: intake at or above the AI level was deemed adequate, while intake below the AI was considered insufficient. In calculating the intake of vitamins and minerals in the diet, only the food consumed by the subjects was considered, excluding any supplementation used. When calculating the daily caloric intake of the subjects, the proportion of energy from proteins, carbohydrates, and total fat was taken into account, along with factors such as age, sex, body weight of the subjects, and physical activity level (PAL).

Two approaches were utilized to calculate the energy requirements of children and adolescents with PWS. The first approach involved using equations recommended by the FAO/WHO/UNU, as indicated in the “Nutrition standards for the population of Poland” in 2020 [25], to calculate total energy expenditure (TEE) in kcal. For boys aged 1–18, the equation:

TEE = 310.2 + 63.3 × W − 0.263 × W^2^ (1)

was employed, while for girls aged 1–18, the equation:

TEE = 263.4 + 65.3 × W − 0.454 × W^2^ (2)

was used, where W represents the reference body weight for a specific sex and age group.

To determine the energy requirement for an individual with PWS receiving GH treatment, 75% of the TEE value was calculated based on the aforementioned equations. Reference body weight for age and sex was obtained from the current Polish standard for body weight, presented as a centile grid dedicated to children of both sexes aged 3–18. This standard was developed as part of the OLA NR13 000206 and OLAF PL0080 programs [20]. The body weight at the 50th percentile for a specific age and sex was considered as the reference body weight. For a child aged 2 years and 8 months, the reference body weight was assumed to be the same as that of a 3-year-old child.

Within the second approach, energy standards were adopted based on the estimated energy requirement (EER) indicated by the current Polish standards for normal body weight, adjusted for sex and age. Starting from the age of 7, the Polish standards also consider energy demand differentiation based on PAL, and from the age of 10, they additionally take gender into account [25]. The PAL values for the subjects were determined through interviews with the caregivers of subjects with PWS, following the guidelines provided by FAO/WHO/UNU:PAL = 1.4 for sedentary individuals (seated work, lack of physical activity); PAL = 1.6 for those with moderate physical activity (sedentary work, low physical activity related to standing, walking); PAL = 1.75 for active individuals (work predominantly involving walking, standing, or sitting combined with regular physical activity of about 1 h per day at moderate intensity, active or moderately active lifestyle); and PAL = 2.0 for very active individuals (regular, demanding physical work or forced activities during free time for over one hour per day, vigorous or highly active lifestyle) [27]. None of the subjects achieved a PAL of 2.0. To determine the actual energy demand for each child with PWS, 75% of the EER value, obtained according to the specified guidelines, was calculated considering the impact of GH treatment on energy demand.

Two approaches were also used to calculate the energy requirements of adults over 18 years of age with PWS. In the first approach, the energy demand was determined using formulas recommended by the Polish standard for the age group of 18–30 years, which allowed the calculation of basal energy expenditure (BEE). For women, the equation used was:

BEE = (14,818 × W) + 486.6, (3)

and for men, it was:

BEE = (15,057 × W) + 692.2(4)

where W represents body weight. All surveyed adults fell into this age category. When calculating BEE for overweight or obese adults with PWS, the calculation was based on their normal body weight, adjusted for their height. For adults with PWS who had a normal body weight, their current measured body weight was used to calculate BEE. The resulting BEE value was multiplied by the PAL value obtained from the interview with the subjects, thereby obtaining the TEE. The effect of GH on energy requirements was taken into account. For individuals receiving GH treatment, 75% of the calculated caloric demand based on the standard was considered as their individual energy requirement, whereas for patients not receiving GH, 50% of the calculated caloric demand based on the norm was adopted [13,15,28].

As part of the second approach, energy standards for adults with PWS were implemented, based on the EER for individuals with normal body weight according to Polish standards. The Polish standard used in this approach includes six levels of PAL (1.4, 1.6, 1.75, 2.0, 2.2, 2.4). PAL values for the subjects were determined through interviews with their caregivers, following the FAO/WHO/UNU guidelines mentioned earlier. For the 19–30 age category, the Polish standard provides four body weight values (55, 65, 75, and 85 kg for men; 45, 55, 65, and 75 kg for women). For adults with PWS who had a normal body weight (BMI within the range of 18.5–24.9), the EER value was selected from the standard based on the body weight that closely matched their current measured weight. However, for subjects with abnormal body weight, the EER calculation was based on body weight within the BMI range of 18.5 to 24.9 kg/m^2^ (a BMI value of 21.7 kg/m^2^ was used as the reference). To determine the actual energy demand for a specific individual with PWS, 75% of the EER was calculated, taking into account the influence of GH treatment. For patients not receiving GH, 50% of the caloric requirement based on the norm was calculated. In addition, the energy requirement was calculated based on the recommendations of Hoffman and Aultman [29]. These recommendations suggest an energy intake of 8–9 kcal/cm of height for individuals with PWS who require weight loss and 10–11 kcal/cm of height for those who need to maintain their current body weight.

All subjects received dietary advice, with the initial step involving calculations of energy intake and DFRs conducted by the consulting team (R.K., K.S., O.P.). For the purpose of publication, the calculation of energy intake and dietary components in the subjects’ DFRs was performed separately by researchers from the University of Life Sciences in Lublin (P.G., K.N.).

## 3. Results

The parents/guardians of the participants indicated their permanent place of residence as follows: a city with over 100,000 inhabitants (*n* = 9; 45%); a city with a population between 20,000 and 100,000 (*n* = 3; 15%); a city with fewer than 20,000 inhabitants (*n* = 2; 10%); and rural areas (*n* = 6; 30%). Regarding the assessment of the household’s financial situation, the distribution was as follows: living in very poor conditions, with not enough for basic needs (no indications); living modestly, managing daily life very economically (no indications); living at an average level, with enough for everyday life but needing to save for significant purchases (*n* = 9; 45%); living well, able to afford a lot without extensive saving (*n* = 9; 45%); living very well, with the ability to afford certain luxuries (*n* = 2; 10%).

By comparing the BMI data obtained from the study group of subjects with PWS to the Polish standard from the OLA/OLAF programs (including subjects up to 18 years of age, and the WHO BMI standard for children under 3 years of age), as well as the WHO classification (for subjects over 18 years of age), the findings revealed that 65% of the subjects had a normal body weight (*n* = 13). Excessive body weight was observed in 25% of the subjects (*n* = 5), with 15% of them falling into the preobesity (overweight) category (*n* = 3) and 10% classified as obese (*n* = 2). Additionally, 10% of the subjects were identified as underweight (*n* = 2). Data on the nutritional status of the surveyed subjects and information on the primary diagnosis, severity of intellectual disability, and comorbidities are summarized in Table 1.

Table 2 shows the diagnosis of nutritional status and the estimated energy requirements of the studied subjects with PWS, calculated on the basis of the Polish standard, in relation to the genetic type of PWS and the degree of intellectual disability, taking into account the status “treated” or “not treated” with GH. The basic anthropometric parameters related to the nutritional status of the subjects (body weight, height, weight–height index) were also given.

Table 3 and Table 4 summarize the assessment of energy intake calculated from the dietary record in relation to the energy norm for the subjects, as well as the results of the assessment of macronutrient intake, taking into account their proportion in the energy supply.

The average energy value of the analyzed dietary records of subjects with PWS was 1482 ± 295 kcal (Table 5). In the group of males, the average energy intake was 1475 ± 247 kcal, and in the group of females, it was 1494 ± 373 kcal. According to the recommended total metabolic rate for adults with low physical activity (PAL 1.4), adult men (18–28 years old, *n* = 6) should consume 2341 kcal, while women (*n* = 2) should consume 1887 kcal [30]. As a result, 100% of men and 50% of women aged 18–28 had insufficient energy intake. Additionally, 83% of boys (*n* = 5) and 80% of girls (*n* = 4) aged 4–17 years also showed insufficient energy intake.

The energy intake deficiency in the study group aligns with the recommended nutritional care for people with PWS. When comparing the energy intake in the dietary records of subjects with PWS to the recommended energy intake based on kcal per cm of height [11], it can be observed that the average energy intake of subjects with PWS who are not overweight (subjects 1, 2, 4–13, 16, 18, 20) was 10.76 kcal/cm of height, which falls within the recommended range of 10–11 kcal/cm of height for non-overweight individuals with PWS. On the other hand, people with PWS who were overweight (subjects 3, 14, 15, 17, 19) had an average energy intake per cm of height of 9.58 kcal/cm, slightly exceeding the recommended energy intake level of 8–9 kcal/cm for overweight or obese individuals with PWS. It is important to note that all subjects received professional dietary consultation to optimize their energy and food intake. The target energy intake, as included in the meal plans prepared for the subjects, was designed based on the aforementioned recommendations [11].

When comparing the energy intake in the DFRs with the TEE calculated on the basis of the FAO/WHO/UNU equations according to the current “Nutritional Standards for the Polish Population” [25], taking into account the adjustment of energy requirements according to GH treatment status (Table 2), it is worth noting that in 12 subjects (60%), the energy intake in the DFRs was lower than the TEE adjusted for GH treatment status, while in eight subjects (40%), it exceeded this norm. The highest excess of energy intake was observed in a 2-years-and-8-months-old child (subject 11) with an excess of 716 kcal and in a subject with class II obesity (case 17) with an excess of 1183 kcal. In the remaining cases, the excess energy intake was small, ranging from 34 to 230 kcal, with an average of 139 kcal.

Comparable results were obtained by comparing the energy intake in the DFRs of PWS subjects with the EER adjusted for GH treatment status. It was found that in 65% of subjects, the energy intake was lower than the EER adjusted for GH treatment status, whereas in only 35% of subjects, the energy intake in the DFRs exceeded the EER adjusted for GH treatment status. The highest excess was observed in a child aged 2 years and 8 months, with an excess of 822 kcal, and in a subject with obesity class II, with an excess of 1127 kcal. In the remaining five cases, the energy intake exceeded the norm by amounts ranging from 3 to 281 kcal, with an average of 125 kcal (Table 6).

The mean protein intake of the study participants was 69.0 ± 17.4 g (Table 5). For all males, the mean intake was 72.2 ± 16.5 g, with a median intake of 74.0 g. For females, the mean protein intake was 64.2 ± 18.7 g, with a median intake of 63.2 g. No substantial differences in protein intake were found between age groups for either males or females. In 75% of the subjects, the total protein content in the DFRs was within the recommended range of 10–20% of the EER for protein (Table 3). One subject had an inadequate protein intake, while four subjects had an excess of this nutrient. Importantly, all subjects met their protein requirements according to the EAR standard, with only one subject reaching 94% of the RDA standard, while the rest exceeded 100%.

The mean fat intake in the DFRs was 45.7 ± 12.4 g. For males, the mean intake was 43.9 ± 9.6 g, with a median intake of 43.5 g (Table 5). For females, the mean fat intake was 48.4 ± 16.0 g, with a median intake of 44.4 g. No noteworthy differences in fat intake were observed between the age groups 2–17 and 18–28 for males. However, there were noteworthy differences for women. In the age group 2–17, the mean fat intake was 43.4 ± 9.6 g. In the age group 18–28, two women had significantly higher fat intakes—44.0 g in a 24-year-old woman and 82.6 g in a 21-year-old woman. Of all the subjects, 30% (*n* = 6) were fat deficient, while 20% (*n* = 4) were fat overconsumers. In addition, 80% of the subjects had a diet characterized by excessive consumption of saturated fats, with an average intake of 146% of the EER.

The mean carbohydrate intake was 220 ± 45 g (Table 5). There were no significant differences in the mean carbohydrate intake between males and females (224 ± 44 g and 213 ± 48 g, respectively). However, differences between age groups were observed for both sexes. In males aged 2–17 years, the average carbohydrate intake was 16% higher compared to the group of males aged 18–28 years. In females, 26% higher carbohydrate consumption was observed in the 18–28 age group. Among the study participants, 10% did not consume enough carbohydrates, defined as 45–65% of the EER, while 20% of the DFRs showed excessive carbohydrate intake (Table 4). It is worth noting that all subjects met the RDA standard for carbohydrate intake (130 g). For fiber, the mean intake was 25.8 ± 5.5 g (Table 5). Differences were observed between sex and age groups. Females consumed approximately 14% more dietary fiber than males (27.8 ± 5.6 g and 24.5 ± 5.3 g, respectively). Among age groups, there were no significant differences in fiber intake for males (23.9 ± 6.4 g for the 2–17 age group and 25.0 ± 4.4 g for the 18–28 age group). In females, however, there was a significant 25% difference between the 2–17 and 18–28 age groups (26.1 ± 4.8 g and 32.9 ± 6.1 g, respectively). Three subjects had a fiber deficiency.

Data on vitamin intake in the DFRs of subjects with PWS are presented in Table 7 and Table 8. Analysis of the vitamin intake in the DFRs of the subjects and the coverage of vitamin requirements shows that the intake of vitamin B1 was correct in 95% of the subjects (195% of the EAR) and vitamin B_3_ and folic acid in 100% of the subjects (166% and 135% of the EAR, respectively). The DFRs covered vitamin B_6_ in 95% of subjects and vitamin C in 90% of subjects (162% and 267% of EAR, respectively). The intake of vitamin B2 was sufficient in 85% of subjects (138% of EAR). In the case of the vitamins mentioned above, their consumption was usually higher in the female group than in the male group (Table 9). No vitamin A deficiency was observed. The mean intake of all participants for this vitamin was 285% of the EAR. Vitamin E intakes were insufficient in 85% of subjects, and vitamin B_12_ intakes were insufficient in 40% of subjects. None of the study participants’ DFRs met the requirements for vitamin D.

There were no differences in the intakes of most minerals between females and males or between age groups (Table 10). The results of the analysis of dietary mineral intakes in the DFRs are presented in Table 11 and Table 12. The DFRs of 100% of the subjects did not provide the appropriate amount of iodine. The mean iodine intake was 41.9 ± 12.7 g for males and 30.7 ± 16.6 g for females (median 38.7 g and 24.9 g, respectively). Most DFRs were deficient in sodium (85% of subjects) and copper (60% of subjects). Calcium intake was deficient in seven subjects (35%). Most or all subjects in all age groups had normal intakes of potassium (15), phosphorus (all), magnesium (17), iron (all), manganese (19), and zinc (17).

## 4. Discussion

There is a lack of Polish data on energy and nutrient intake in patients with PWS. Comparisons with studies conducted in populations with PWS from other countries (limited studies available in the world literature) are limited due to differences in the calculation of energy standards for PWS and differences in national nutritional standards.

Upon analyzing the nutritional status of the subjects with PWS, it was observed that five subjects (25%) exhibited excessive body weight, including two subjects who were classified as obese (10%). This indicates that the subjects with PWS receive effective nutritional supervision, which, combined with the beneficial effects of GH treatment, enables the majority of subjects to prevent pathological weight gain. The two subjects with class II obesity were not treated with GH due to medical contraindications. The prevalence of obesity in PWS is estimated to be around 40% in children and between 82% and 98% in adults. However, individuals with PWS consistently exhibit disturbed body composition, characterized by a relatively high fat mass and low lean mass [10]. Therefore, the use of BMI to assess the nutritional status in PWS is limited. According to Scheimann et al., individuals with PWS should be considered obese regardless of their measured body weight, as the key aspect of obesity is the excess fat relative to nonfat mass [9]. The underlying cause of the lean mass deficit and the imbalance between lean mass and fat mass in individuals with PWS remains uncertain. It is not known whether the primary issue is excessive lipogenesis or decreased muscle/bone formation [9].

Before discussing the results of nutritional status assessment, the justification for the use of selected standards is necessary. Based on the available literature, the use of population percentile charts to assess the nutritional status of children with PWS treated with GH is justified. GH treatment normalizes the growth process and has a positive impact on body composition, increasing skeletal muscle mass and bone density, and reducing fat mass. These effects promote higher levels of physical activity, increase energy demand, and lead to favorable changes in metabolism [1,31,32].

When discussing the results of energy intake by subjects with PWS, the authors of the reported studies refer to energy recommendations for PWS expressed in kcal/cm of height. This approach is used due to limitations encountered when estimating energy demand in PWS using traditional methods, which do not account for the unique patterns of body weight growth and gain in this population [33]. It has been suggested that for PWS patients requiring weight loss, the energy supply should be set at 8–9 kcal/cm, while for patients not requiring weight loss, the recommended energy intake is 10–11 kcal/cm of height [11,29]. However, Scheimann et al. and Stadler propose slightly different energy intake norms for PWS: 7 kcal/cm for weight loss and 8–11 kcal/cm for weight maintenance [9,33]. Our own research revealed that the average energy intake in the DFRs of non-overweight subjects with PWS was 10.76 kcal/cm of height, which fell within the recommended range. In contrast, subjects with PWS who were overweight exhibited an average energy intake per cm of height of 9.58 kcal/cm, exceeding the recommended energy intake level of 8–9 kcal/cm for overweight or obese individuals with PWS. In a study conducted by Meade et al. in Ireland, the energy intake in children with PWS aged up to 5 years was 9 kcal/cm of height, while in the 5–12-year age group, it was 8.3 kcal/cm of height [34]. It is worth noting that the referenced energy standards expressed in kcal/cm of height may be overestimated for physically inactive individuals with PWS and underestimated in the case of GH treatment. In the population of healthy children, maintaining a normal body weight typically ensures an energy intake level of 11–14 kcal/cm of height [9]. The recommended energy intake for weight loss in adolescents and adults with PWS is 800–1100 kcal/day, while for children, it is 600–800 kcal/day [9,33]. Considering this recommendation, it is important to note that the overweight or obese subjects with PWS in our study exceeded the recommended energy intake. In the absence of nutritional supervision, individuals with PWS may consume energy intakes as high as 5167 kcal per day [33].

When comparing the results of our own research on energy intake in the PWS study group to findings from other authors, the Canadian study by Mackenzie et al. on young subjects with PWS (*n* = 23, mean age 8.5) provides valuable insights [35]. The study compared the energy intake of patients with PWS to that of healthy subjects and found significantly lower energy intake in PWS patients (1523 kcal vs. 2000 kcal, *p* < 0.001), equivalent to 11.3 kcal/cm and 13.4 kcal/cm, respectively. In our own research, the average energy intake in the PWS group was 1518 kcal, similar to the results obtained by Mackenzie et al. in the patient group, and 10.47 kcal/cm of height, slightly lower than in Mackenzie et al. findings [35]. Another study by Rubin et al. on the dietary quality of young subjects with PWS (*n* = 32, mean age 10.8) compared them to obese individuals without a PWS diagnosis (*n* = 48) [28]. The study revealed that subjects with PWS consumed fewer calories, carbohydrates, and sugars compared to obese youth without PWS, but they consumed more vegetables. Energy intake in the PWS group was 14% lower than in the non-PWS group, while the proportions of dietary macronutrients in the DFRs were similar. In a Norwegian study by Lindmark et al., dietary component intake in children with PWS (*n* = 7) was compared to the Nordic Nutritional Recommendations (NNR) and a control group of healthy subjects [36]. The study showed that the energy intake in PWS patients compared to the EER determined according to the NNR was 61–77% of this norm. However, when compared to the energy intake in the group of healthy children, the energy intake in PWS patients was 60% in 2-year-olds and 66% in 4-year-olds. In an Irish study by Meade et al., which included 19 children with PWS (mean age 7.6), the energy intake in the age group up to 5 years old was 722 kcal, equivalent to 9 kcal/cm of height (72–112% EAR) [34]. For the 5–12 age group, the average energy intake was 1203 kcal, i.e., 8.3 kcal/cm of height (41–82% EAR).

An adequate energy value in the diet is a crucial aspect of PWS treatment to prevent excessive weight gain. However, it is important to note that restrictive reduction diets may increase the risk of inadequate nutrient intake [35]. This observation served as an important premise for conducting our own research. It is known that individuals with PWS have lower energy requirements compared to healthy individuals, primarily due to lower lean mass relative to body weight and reduced energy expenditure during physical activity [37]. In PWS, excessive body weight is often the result of an overly high energy intake (above the total metabolic rate) [38]. The analysis of DFRs in the majority of the examined subjects indicated energy deficiencies. This suggests that weight maintenance and gains in PWS occur at lower energy intake levels. When discussing the nutritional supervision of people with PWS, attention is often drawn to the habit of eating meals in secret [39], as reported by parents in the food diaries analyzed in our own research. It is common for adult patients with PWS to admit to the frequent consumption of snacks and calorie-dense foods outside regular meals, while children often eat in secret from their parents [40]. One contributing factor to weight gain in PWS may be the preference for sweet tastes and calorie-dense foods, as demonstrated in various studies [41]. It appears that individuals with PWS tend to seek out such foods outside of the nutrition plans implemented by their caregivers.

Discussing results on energy intake in patients with PWS, it should be noted that the disease itself and GH treatment impact energy demand. The energy demand for the subjects with PWS in this study was estimated considering the assumption from Polish pediatric endocrinology that PWS reduces daily caloric demand by 50% compared to healthy children of similar body weight. However, treatment with GH is known to increase energy demand to 75% of the demand for healthy children of similar body weight [13].

Before the intake of nutrients is discussed, we wish to state that the EAR was chosen as the reference level, as it is used in Polish nutritional standards to assess the likelihood of insufficient or excessive intake by individuals or groups [42]. When discussing the intake of macronutrients in the diet, it is important to note that in the majority of cases, the intake of protein, total fat, and carbohydrates was adequate. However, excessive intake of saturated fatty acids (SFA) was found in the majority of subjects. In our own research, protein intake in the DFRs of 19 out of 20 subjects exceeded the RDA standard, with an average achievement of 180% of the norm in the study group, as well as the EAR norm, with an average achievement of 221% of the norm, despite reduced energy intake. The correct protein intake in PWS patients, as demonstrated in this study, is important in relation to their lean mass deficiency. In the treatment of obesity in non-PWS individuals, excess fat mass provides relative protection against lean mass loss. This means that people with a higher excess of fat mass tend to lose relatively less lean mass compared to those with a smaller excess of fat mass [9]. However, this pattern does not apply to patients with PWS. Therefore, maintaining adequate protein intake, even at the level of 1.5 g/kg, is particularly important in the nutritional management of PWS patients [9,43]. Protein in the nutrition of patients with PWS should be of high biological value, meaning it should contain all essential amino acids. Complex carbohydrates should be preferred, while fat should be limited to what naturally occurs in food [33]. Attention is drawn to the potential role of adequate protein intake in enhancing satiety, which is greater than that of carbohydrates or fats [9,43]. Proper protein intake is also important in the case of insufficient calcium supply observed in the tested DFRs, as a high-protein diet promotes the excretion of calcium in urine [44]. In the study by Mackenzie, protein intake was deficient in only 1 out of 22 PWS subjects [35]. In the study by Rubin et al., protein intake in the PWS group and the obese group without PWS was similar, averaging around 0.8 g/kg body weight [28]. Mackenzie et al. demonstrated that protein intake exceeded the EAR norm in almost all subjects with PWS [35].

In our own research, fat intake was insufficient (below 20% EER) in 6 out of 20 subjects, adequate in 10 subjects, and exceeded the recommended norm (35% EER) in only 4 subjects. A Norwegian study by Lindmark et al. on a group of children with PWS showed very low fat intake, at 24% of energy (E) in 2-year-olds and 25% E in 3- and 4-year-olds, significantly below the recommended levels [36]. In our research, carbohydrate intake was insufficient (below 45% EER) in 2 out of 20 subjects, adequate in 14 subjects, and exceeded the norm (65% EER) in only 4 subjects. However, the intake of dietary fiber was insufficient in only three subjects. In Mackenzie’s study, fat intake was insufficient in 7 out of 22 subjects, carbohydrate intake in 1 out of 22 subjects, and fiber intake was insufficient in 17 out of 22 subjects [35]. Our findings regarding fiber intake may have been influenced by the timing of the research, as it was conducted during the spring and summer months when there is a greater availability of vegetables and fruits in Poland. The results may have been different if the study was conducted in the winter months. A study by Miller et al. conducted on children with PWS found that carbohydrate and protein intake were at the correct level, but there was a deficiency in fat and dietary fiber intake [45]. Similarly, in a group of subjects with PWS from Ireland, deficient fiber intake was demonstrated [34]. In Norwegian studies by Lindmark et al. [36], fat intake in young children with PWS aged 2–4 years was low, at 24–25% E (the Polish standard recommends a fat intake of 35–40% E for the age group of 1–3 years and 20–35% E for the age group of 4–18 years), protein intake was higher than the norm (similar to our own research), and carbohydrate intake corresponded to the norm (although, in our own research, almost half of the subjects had lower carbohydrate intake than the norm). Referring to the results that indicate insufficient fat intake in some of the subjects, including our own research and studies by other authors [36], it should be noted that parents of children with PWS often restrict the consumption of fats. They believe that this is an effective strategy to lower the energy content of the diet and prevent obesity.

The standard approach to nutritional management in PWS involves implementing a balanced, energy-restricted diet. The recommended energy intake for individuals with PWS depends on their GH treatment status. It is worth noting that the literature acknowledges the lack of clear guidelines specifically addressing energy intake recommendations for individuals with PWS. Instead, a general recommendation is “to lower the energy intake to maintain a healthy body weight” [8]. However, Alsaif et al. argue that this approach fails to consider the challenges posed by hyperphagia, impaired satiety, and food-seeking behaviors that complicate nutritional management in PWS [8]. As previously mentioned, in the field of Polish endocrinology, it is assumed that the energy demand for patients with PWS undergoing GH treatment is around 75% of the requirement for healthy individuals based on standard calculations. For those not receiving GH treatment, the estimated energy demand is approximately 50% [13].

Regarding macronutrient ratios, Miller et al. demonstrated that implementing a low-energy diet with a fat content of 30% E, carbohydrates at 45% E, and protein at 25% E, along with a dietary fiber intake of at least 20 g per day, resulted in a more significant reduction in body fat content among PWS children aged 2–10 years (*n* = 63) compared to those who only reduced their energy intake without modifying the proportions of macronutrients [45]. These ratios are currently recommended as the standard approach in PWS management. It is also recommended to align the PWS diet with the Mediterranean diet model, incorporating small and frequent meals throughout the day to help control hyperphagia [32,46].

In our own research, we followed the Polish standard, which recommends that protein provides 10–20% E, fats contribute 20–35% E (including SFA up to 6% E), and carbohydrates supply 45–65% E. Upon analyzing the food diaries, it was observed that the majority of participants met the protein intake recommendations (only one subject had insufficient intake), while six and two subjects did not meet the norms for fats and carbohydrates, respectively. The intake of SFA exceeded the recommended limit in 16 subjects. It appears that the approach adopted by caregivers is to reduce fat and carbohydrate intake while maintaining protein intake at a relatively high level. In the study by Rubin et al., dietary fat provided 28.9% E, carbohydrates accounted for 54.0% E, and protein contributed to 18.9% E in the diets of individuals with PWS [28].

Referring to the obtained results regarding the intake of vitamins and minerals in the diet, it is important to highlight that individuals with PWS who receive a diet with an energy deficit may have insufficient intake of essential vitamins and minerals. Furthermore, low sun exposure should be taken into account, particularly in individuals with hypopigmentation, which can further contribute to vitamin D deficiencies. Scheimann et al. suggest that all individuals with PWS should receive vitamin and mineral supplementation, with careful monitoring of trace elements, especially iron, and fat-soluble vitamins, to prevent potential overdosing [9]. Stedler also emphasizes that prolonged caloric restrictions can predispose individuals with PWS to deficiencies in vitamins and minerals, necessitating the need for supplementation [33]. Currently, there is a lack of randomized placebo-controlled clinical trials on supplementation in PWS. Therefore, conducting such studies would be highly justified to assess the clinical benefits of off-label supplementation and establish evidence-based guidelines for individuals with PWS [47].

Based on our own research, the uptake of vitamins and mineral components of the diet without supplementation was assessed in order to estimate the connection between the caloric deficit and the quality of the diets of PWS patients. Our own research did not show a deficiency of the B-group vitamins (B_1_, B_2_, B_3_, B_6_, B_12_) in all or in the vast majority of subjects. The calculated intake of over 90 percent of EAR or AI probably stemmed from the appropriate consumption of cereal and protein products. The intake of folic acid in 19 out of 20 subjects exceeded the EAR norm (the average realization of the EAR was 175% in the studied group). A deficient intake of vitamin C was found only in two subjects and the vitamin A intake in all the subjects met the EAR, whereas the highest intake deficiencies concerned vitamins D and E. A deficient intake of vitamin D was found in all the subjects: the average supply of this vitamin was only 2.93 μg in the studied group. In contrast, a deficient intake of vitamin E was found in 17 out of 20 subjects.

Similar findings were reported by Mackenzie et al., who found that all Canadian PWS patients included in their study did not meet the requirements for vitamin D (vitamin E intake was not assessed in this study) [35]. However, the intake of vitamins B_6_ and B_12_ was sufficient in all (22/22) and the majority (21/22) of the participants, respectively, which aligns with the results of our own research. On the other hand, the intake of folic acid met the norm in 15 out of 22 subjects, indicating a slightly lower coverage compared to our study. In terms of vitamin A intake, the norm was met in 18 out of 22 subjects according to the Canadian study [35]. In the study by Rubin, only 2 out of 32 subjects met the norm for vitamin D intake, while the intake of water-soluble vitamins (B_1_, B_2_, B_3_, B_6_, B_12_) was sufficient in the vast majority of participants (26/32, 30/32, 27/32, 29/32, 25/32, respectively) [28]. Similarly, the Irish study by Meade et al. showed suboptimal intake of vitamin D in a group of 19 children with PWS [34]. The Norwegian study by Lindmark et al. revealed sufficient intake (without supplementation) of vitamins and minerals (similar to our own research), with the greatest deficits observed in vitamin D intake (ranging from 26% to 38%) (similar to our findings) [36].

The analysis of dietary mineral intake revealed deficiencies in iodine intake (100% of subjects) and sodium (17 out of 20, which is 85% of subjects). It is important to note that the data on sodium and iodine intake may be underestimated because parents did not include information about adding salt to food in their food diaries. In Poland, the prevention of iodine deficiency includes the mandatory iodization of table salt, as well as infant milk and mineral waters [48]. The safe intake levels of sodium recommended by EFSA are 1.1 g/day for children aged 1–3 years, 1.3 g/day for children aged 4–6 years, 1.7 g/day for children aged 7–10 years, and 2.0 g/day for children aged 11–17 years, respectively. Based on these guidelines, it can be concluded that two subjects from the age group of 2–17 years in the study exceeded the recommended sodium intake, with an excess of 100 mg for males and 500 mg for females. In a Canadian study by Mackenzie et al., the analysis of food diaries of children with PWS showed that sodium intake was deficient in only 2 out of 22 subjects, while 17 out of 22 subjects with PWS had sodium intake exceeding the UL standard [35]. However, iodine intake was not assessed in this study.

In the study group, 35% of subjects (7/20) had a calcium deficiency in their diet. It is important to highlight that children with PWS are more prone to developing osteopenia and osteoporosis due to factors such as reduced muscle mass, hypogonadism, hypotonia, and lower levels of physical activity. In the study by Meade et al., more than half of the children with PWS under 5 years of age and 62% of children over 5 years of age met the AI standards for calcium [34]. However, in the study by Mackenzie et al., calcium intake was deficient in 12 out of 22 subjects [35]. Similar deficiencies in calcium (and vitamin D) intake were noted in the study by Bakker et al., which focused on children and adolescents with PWS [15]. On the other hand, in the study by Rubin et al., subjects with PWS had a higher coverage of calcium demand (5 out of 27) compared to the control group of individuals with obesity but without PWS (1 out of 47) when considering supplementation [28]. However, even with supplementation, the coverage of calcium demand was only 18.5% of the subjects. Magnesium intake in our own research showed deficiencies in only 3 out of 20 subjects with PWS. In the study by Mackenzie, 5 out of 22 subjects were found to have deficiencies in magnesium intake (note that these studies assessed dietary component intake without considering supplementation) [35]. Regarding other minerals, our own research revealed the following results: potassium intake was deficient in 5 out of 20 subjects, while phosphorus and iron intake met the recommended norms in all subjects. For zinc, the EAR norm was met in 17 out of 20 subjects, while manganese intake was deficient in only one subject. In terms of copper intake, our own research showed deficiencies in 12 out of 20 subjects (60% of respondents). In contrast, Mackenzie et al. reported significantly higher copper intake (0.7 mg/1000 kcal) in the study group of children with PWS compared to the control group of healthy peers, although the coverage of the norm in their study group was not provided [35]. In the study by Rubin et al., iron intake was adequate in all subjects with PWS (32/32), and more subjects met the norm compared to the obese control group without PWS [28]. In the Irish study by Meade et al., patients with PWS showed suboptimal intake of calcium, iron, and zinc [34]. In the study by Lindmark et al. conducted on young children with PWS, iron intake ranged from 84% to 96% of the norm, indicating suboptimal levels, while calcium intake reached the recommended level [36].

In summary, based on our own research, most subjects exhibited deficiencies in the intake of iodine, sodium, and copper, while the intake of other mineral components of the diet met the recommended standards for the majority of subjects (including calcium, potassium, phosphorus, magnesium, iron, manganese, and zinc). When considering deficiencies in the intake of dietary components in patients with PWS, as indicated in the literature (such as calcium, vitamin D, and iron), it is worth noting that in our study population of Polish subjects with PWS, deficits were observed in the intake of vitamin D and calcium in 35% of the subjects, whereas none of the subjects had deficiencies in iron intake.

This study has several limitations. Firstly, the study group was relatively small, and there was a wide range of ages among the participants. However, it is worth noting that similar studies in the field have also employed small study groups. For example, the Canadian study by Mackenzie included 22 subjects with PWS, Rubin et al. examined the diet quality of 32 subjects with PWS, and Lindmark et al. conducted a study on dietary intake with a group of six children with PWS [28,35,36]. To address the challenge of the significant age range among the participants, we accounted for age-specific consumption norms for each analysis.

Despite the fact that the sample size was small and exhibited diversity in terms of age, these results are the first of their kind in Poland and will serve as an important starting point for future research. It should be noted that accessing such patients poses significant challenges, resulting in a reduced study population size. The authors consider these reported studies as a pilot for a broader research project aimed at assessing the quality of the diet in a representative sample of Polish children, adolescents, and young adults with PWS.

The authors acknowledge that current recommendations suggest accounting for supplementation in the nutritional management of PWS [49]. However, the primary aim of the authors was to assess the actual intake of dietary components without supplementation in the diets of Polish patients with PWS, in order to formulate recommendations for supplementation tailored to the specific nutritional needs of Polish patients. Furthermore, we plan to assess the impact of supplementation on the intake of dietary components in the study group and determine to what extent it can help correct nutritional deficits observed in the dietary component intake from DFRs.

Another limitation of the study was that the calculations of energy intake and dietary components relied on self-completed food diaries by the parents and guardians of subjects with PWS, which may introduce potential bias. It is important to note that completing food diaries requires full commitment, and there can be instances of insufficient recording of meals and changes in eating behavior, which are common mistakes when using this type of food record [50]. However, in the case of the caregivers of subjects with PWS who are trained in nutritional supervision, it is likely that this limitation does not apply. Despite its known shortcomings, dietary records remain a valuable tool for assessing the consumption of dietary components. Furthermore, it should be acknowledged that in the case of PWS, there may be an underestimation of nutrient intake, particularly caloric intake, due to children accessing food without the knowledge of their parents or guardians. This factor should be taken into consideration when interpreting the results.

One advantage of this research is the integration of the analysis of dietary intake levels with the anthropometric and clinical status of the surveyed subjects. Furthermore, the surveyed subjects benefited from targeted and individualized dietary interventions based on the data and parameters obtained during the study. All parents or guardians of the examined subjects received personalized dietary advice aimed at addressing any irregularities in their diet. This dietary advice was provided through email, with the option for direct communication via electronic means with a dietician for real-time support, if desired by the individuals.

Due to the deliberate sample selection, the conclusions drawn from this research are limited to the surveyed population. The research findings reaffirm that despite advancements in medical pharmacotherapy dedicated to PWS, the caregivers of patients with PWS face an ongoing challenge in optimizing the metabolic profile of patients through proper nutrition. This includes maintaining a restrictive yet tailored energy intake while ensuring the intake of essential nutrients. Building upon the experiences of caregivers described in a previous publication by Kowal et al., it is evident that obesity prevention in PWS requires a multifaceted approach, encompassing not only dietary interventions but also behavioral interventions [18].

## 5. Conclusions

The energy intake in the study group corresponded to the principles of nutrition in PWS, taking into account the status of GH treatment. Despite the reduced caloric intake, the majority of subjects met the requirements for most macro- and micronutrients in their diet. Most subjects had appropriate proportions of macronutrient consumption, although there was a tendency for excessive intake of SFA, which aligns with the typical dietary patterns in the Polish population.

The main issue identified in the study group was the deficient intake of vitamin D, sodium, and iodine. It should be noted that the deficits in sodium and iodine intake observed in the dietary record analysis may not accurately reflect the actual intake, as the records do not account for salting dishes with table salt. In Poland, table salt is mandatorily enriched with iodine. Therefore, we do not consider the deficits in sodium and iodine intake as indications for dietary correction. However, it is highly recommended to use nutritional supplements for vitamin D in individuals with PWS in Poland.

All parents/guardians of the subjects included in this research program received personalized dietary advice, with a focus on correcting specific deficiencies in the intake of dietary components identified in each patient. Guidelines for the health supervision of individuals with PWS should take into account the dietary practices of families caring for people with PWS, with an emphasis on preventing and addressing nutritional irregularities associated with the use of a reduced diet in the management of PWS patients.

## Figures and Tables

**Table 1:** Characteristics of the studied participants.

Distribution	*n*	%
Gender		
Male	12	60
Female	8	40
Level of intellectual disability (ID)		
No ID	4	20
Mild	9	45
Moderate	4	20
Severe	2	10
No data ^1^	1	5
Genetic type of PWS		
Type I	12	60
Type II	7	35
Imprinting defects	0	0
No data	1	5
Assessment of the nutritional status ^2^		
Underweight	2	10
Normal weight	13	65
Preobesity (overweight)	3	15
Obesity ^3^	2	10
Comorbidities ^3^		
No comorbidities	4	20
Hypothyroidism	4	20
Hypogonadotropic hypogonadism	1	5
Arterial hypertension	2	10
Sleep apnea	2	10
Behavioral/emotional disorders	3	15
Insulin resistance	3	15
Defect of vision	4	20
Adrenal insufficiency	3	15
Allergy	3	15
Epilepsy	1	5
Asthma	1	5
Spine deformities	4	20
Cryptorchidism	1	5
Autism spectrum disorder	3	15

^1^ The level of intellectual disability in this child has not yet been determined at the time of data collection due to her age. ^2^ Polish standards were used to assess the nutritional status of the subjects with PWS aged 3–18 (*n* = 12). In the case of adults (≥18 years) (*n* = 8), nutritional status was assessed using the WHO categorization of BMI: BMI below 18.5—underweight; 18.5–24.9—normal weight; 25.0–29.9—preobesity; 30.0–34.9—obesity class I; 35.0–39.9—obesity class II; above 40—obesity class III [22]. The nutritional status of a child aged 2 years and 8 months was assessed using the WHO standard dedicated to the assessment of the physical development of children aged 0–5 years (BMI percentile chart) [21]. ^3^ The sum of the percentage numbers does not add up to 100 because each percentage represents the prevalence of a specific disease among the subjects. Data on comorbidities were obtained through interviews with the caregivers of subjects with PWS and by analyzing the patients’ medical records. It is important to note that obesity, although present in two subjects, was not included in the list of comorbidities as it is considered the underlying disease in PWS.

**Table 2:** Nutritional status in the studied group in relation to basic clinical data and the energy requirements ^1^.

No.	Age	Sex	Genetic Type of PWS	ID Severity	GH Treatment	Nutritional Status (OLA/OLAF, BMI)	TEE for Children and Adolescents According to FAO/WHO/UNU ^2^ (kcal)	EER According to the Polish Standard for Children and Adolescents ^3^ (kcal)	TEE for Adults According to the Polish Standard (kcal)	EER According to the Polish Standard for Adults (kcal)
1.	21	M	I	Moderate	Yes	Normal	n.a. ^4^	n.a.	2746 * (2059)	2650 * (1988)
2.	18	M	II	Mild	Yes	Underweight	2605	2550	n.a.	n.a.
3.	15	F	I	Mild	Yes	Preobesity	1837	1575	n.a.	n.a.
4.	13	M	I	Mild	Yes	Normal	2057	2250	n.a.	n.a.
5.	11	M	I	No ID	Yes	Normal	1752	1763	n.a.	n.a.
6.	11	F	I	No ID	Yes	Normal	1567	1838	n.a.	n.a.
7.	11	M	II	Mild	Yes	Normal	1752	1763	n.a.	n.a.
8.	4	F	II	No ID	Yes	Normal	932	1050	n.a.	n.a.
9.	10	M	II	Mild	Yes	Normal	1618	1763	n.a.	n.a.
10.	19	M	I	Mild	Yes	Normal	n.a.	n.a.	2939 * (2204)	2900 * (2175)
11.	2 y. 8 m.	F	No data	No data	Yes	Normal	856	750	n.a.	n.a.
12.	25	M	I	Mild	No	Normal	n.a.	n.a.	2608 ** (1304)	2450 ** (1225)
13.	6	F	I	No ID	Yes	Normal	1078	1050	n.a.	n.a.
14.	24	F	II	Severe	Yes	Preobesity	n.a.	n.a.	1735 * (1301)	1800 * (1350)
15.	23	M	II	Moderate	Yes	Preobesity	n.a.	n.a.	2672 * (2004)	2650 * (1988)
16.	7	M	I	Mild	Yes	Normal	1258	1575	n.a.	n.a.
17.	21	F	I	Mild	No	Obesity class II	n.a.	n.a.	1937 ** (968)	2050 ** (1025)
18.	12	F	II	Moderate	Yes	Normal	1674	1575	n.a.	n.a.
19.	28	M	I	Severe	No	Obesity class III	n.a.	n.a.	2214 **(1107)	2100 ** (1050)
20.	15	M	I	Moderate	Yes	Underweight	2347	2250	n.a.	n.a.

^1^ For additional data including body mass, height, BMI, and PAL, see Table S1. ^2,3^ The values provided represent 75% of the energy requirement for each subject’s gender and age, as all subjects included in this study are being treated with growth hormone (GH). ^4^ n.a.—non applicable. * For adults receiving GH treatment, the energy demand is modified to 75% of the standard calculated demand (applies to adult subjects: 1, 10, 14, 15) [13]. ** For adults not receiving GH treatment, the energy demand is indicated at 50% of the standard calculated demand (in brackets) (applies to adult subjects: 12, 17, 19) [13]. Abbreviations: ID—intellectual disability; EER—estimated energy requirement; TEE—total energy expenditure.

**Table 3:** Assessment of energy and macronutrient intake in subjects with PWS–part 1 ^1^.

Energy			Protein						Fat		SFA	
No.	Daily Energy Intake (kcal)	Daily Energy Intake in per cm of Height ^2^ (kcal)	Average Intake (g)	Assessment of the Implementation of EER	RDA (g)	RDA Coverage (%)	EAR (g)	EAR Coverage (%)	Average Intake (g)	Assessment of the Implementation of EER	Average Intake (g)	Assessment of the Implementation of EER
1	1615	9.2 (10–11)	84.7	adequate	61.2	138	56.4	150	34.7	insufficient	11.5	adequate
2	1091	6.2 (1–11)	48.2	insufficient	51.1	94	41.5	116	31.3	insufficient	11.1	adequate
3	1427	8.8 (8–9)	72.5	adequate	69.5	104	53.0	137	38.5	adequate	11.9	excessive
4	1763	11.3 (10–11)	83.3	adequate	50.8	164	38.8	215	41.1	insufficient	16.5	excessive
5	1786	11.7 (10–11)	77.5	adequate	39.1	198	29.8	260	56.2	adequate	15.8	excessive
6	1735	11.7 (10–11)	56.2	adequate	43.2	130	33.0	170	51.6	adequate	10.6	adequate
7	1541	11.4 (10–11)	67.6	adequate	35.2	192	26.9	251	41.0	adequate	13.0	excessive
8	1053	11.6 (10–11)	47.8	adequate	14.4	332	10.3	463	34.2	adequate	10.8	excessive
9	1441	10.5 (10–11)	54.0	adequate	31.0	174	23.7	228	35.3	insufficient	8.9	adequate
10	1776	9.8 (10–11)	82.4	adequate	68.4	120	63.1	131	45.6	insufficient	17.5	excessive
11	1572	18.3 (10–11)	67.6	excessive	13.7	494	11.3	596	57.3	excessive	18.5	excessive
12	1506	9.4 (10–11)	61.6	excessive	47.7	129	51.4	120	53.3	excessive	14.0	excessive
13	967	8.9 (10–11)	37.4	adequate	19.7	190	15.0	249	33.9	adequate	9.4	excessive
14	1532	10.0 (8–9)	58.8	adequate	52.5	112	42.6	138	44.0	adequate	14.2	excessive
15	1564	9.0 (8–9)	81.4	adequate	67.1	121	54.4	150	51.2	adequate	15.1	excessive
16	1213	10.2 (10–11)	51.1	adequate	19.4	264	18.1	283	34.7	insufficient	12.2	excessive
17	2152	12.9 (8–9)	98.7	excessive	62.5	158	50.7	195	82.6	excessive	19.1	excessive
18	1519	9.5 (10–11)	74.3	adequate	45.0	165	42.0	177	44.8	adequate	16.1	excessive
19	1186	7.2 (8–9)	70.5	excessive	61.0	116	49.5	142	41.3	excessive	14.5	excessive
20	1921	11.8 (10–11)	104.2	adequate	52.9	197	40.4	258	60.8	adequate	23.3	excessive

^1^ For additional data including the proportion of macronutrients in the energy supply, SF, and fiber intake standards, see Table S2. Concerns also Table 3. ^2^ The recommended energy intake in kcal/cm of height, adjusted according to the subjects’ nutritional status, is provided in brackets. It was assumed that individuals with PWS and normal body weight should have an energy demand of 10–11 kcal/cm to maintain their body weight, while those with excessive body weight should aim for 8–9 kcal/cm [11]. Abbreviations: EAR—estimated average requirement; EER—estimated energy requirement; RDA—recommended dietary allowance; SFA—saturated fatty acids.

**Table 4:** Assessment of energy and macronutrient intake in subjects with PWS–part 2.

Carbohydrates			Fiber		
No.	Average Intake (g)	Assessment of the Implementation of EER	Average Intake (g)	AI Coverage (%)	Assessment of the Implementation
1	256	adequate	31.7	127	adequate
2	159	insufficient	16.4	66	insufficient
3	207	adequate	23.1	110	adequate
4	277	adequate	24.1	127	adequate
5	252	adequate	21.5	113	adequate
6	276	adequate	35.2	185	adequate
7	238	adequate	25.9	136	adequate
8	153	adequate	24.8	177	adequate
9	237	adequate	22.3	139	adequate
10	271	adequate	27.2	109	adequate
11	208	excessive	21.7	217	adequate
12	206	excessive	20.1	80	insufficient
13	139	adequate	25.6	183	adequate
14	240	excessive	28.6	114	adequate
15	209	insufficient	29.8	119	adequate
16	186	adequate	22.7	142	adequate
17	263	excessive	37.2	149	adequate
18	218	adequate	26.4	139	adequate
19	143	adequate	18.4	74	insufficient
20	256	adequate	33.4	159	adequate

**Table 5:** Comparison of energy intake and dietary macronutrients across sex and age groups.

	All Subjects	All Males	All Females	Males 2–17 Years Old	Females 2–17 Years Old	Males 18–28 Years Old	Females 18–28 Years Old
Energy (kcal)	1482 ± 295	1475 ± 247	1494 ± 373	1493 ± 253	1379 ± 304	1456 ± 264	1842 ± 438
Protein (g)	69.0 ± 17.4	72.2± 16.5	64.2 ± 18.7	73.0 ± 14.4	59.3 ± 14.7	71.5 ± 14.4	78.8 ± 19.9
Fat (g)	45.7 ± 12.4	43.9 ± 9.6	48.4 ± 16.0	44.9 ± 11.0	43.4 ± 9.6	42.9 ± 8.8	63.3 ± 27.3
SFA (g)	14.2 ± 3.6	14.5 ± 3.7	13.8 ± 3.7	15.0 ± 4.9	12.9 ± 3.6	14.0 ± 2.4	16.6 ± 3.5
Carbohydrates (g)	220 ± 45	224 ± 44	213 ± 48	241 ± 31	200 ± 49	208 ± 51	251 ± 16
Fiber (g)	25.8 ± 5.5	24.5 ± 5.3	27.8 ± 5.6	23.9 ± 6.4	26.1 ± 4.7	25.0 ± 4.4	32.9 ± 6.1

**Table 6:** Energy intake in DFRs and comparison with total energy expenditure (TEE) and estimated energy requirement (EER) adjusted for growth hormone (GH) treatment status (EER).

No.	Daily Energy Intake (kcal)	TEE Adjusted for GH Treatment Status (kcal)	Excess Energy Intake over TEE Corrected for GH Treatment (kcal)	EER Adjusted for GH Treatment Status (kcal)	Excess Energy Intake over EER Corrected for GH Treatment (kcal)
1	1615	2059	--	1988	--
2	1091	2605	--	2550	--
3	1427	1837	--	1575	--
4	1763	2057	--	2250	--
5	1786	1752	34	1763	23
6	1735	1567	168	1838	--
7	1541	1752	--	1763	--
8	1053	932	121	1050	3
9	1441	1618	--	1763	--
10	1776	2204	--	2175	--
11	1572	856	716	750	822
12	1506	1304	202	1225	281
13	967	1078	--	1050	--
14	1532	1301	230	1350	182
15	1564	2004	--	1988	--
16	1213	1258	--	1575	--
17	2152	968	1183	1025	1127
18	1519	1674	--	1575	--
19	1186	1107	79	1050	136
20	1921	2347	--	2250	--

**Table 7:** Dietary intake of selected vitamins in subjects with PWS–part 1 ^1^.

	Vitamin B_1_		Vitamin B_2_		Vitamin B_3_		Vitamin B_6_		Folic Acid	
No.	Average Intake (mg)	EAR Coverage (%)	Average Intake (mg)	EAR Coverage (%)	Average Intake (mg)	EAR Coverage (%)	Average Intake (mg)	EAR Coverage (%)	Average Intake (µg)	EAR Coverage (%)
1	1.2	170	2.1	162	22.5	177	2.5	167	4197	167
2	1.0	142	1	77	11.5	90	0.9	60	232	93
3	1.1	164	1.3	93	13.6	112	1.7	131	258	103
4	1.2	194	1.9	173	15.5	139	1.9	158	270	129
5	1.1	206	1.0	91	17.8	185	1.7	142	278	133
6	1.4	278	1.7	155	10.2	112	1.5	125	397	189
7	1.0	188	1.4	127	16.3	169	1.7	142	327	156
8	0.7	198	1.0	167	12.6	198	1.7	283	335	305
9	0.9	176	0.9	113	13.7	148	1.4	156	288	180
10	1.3	184	1.8	138	18.6	146	2.1	140	333	133
11	1.1	332	2.1	420	12.9	216	1.6	320	326	362
12	0.6	85	1.4	108	15.1	119	1.4	93	292	117
13	1.1	259	0.5	83	10.4	136	1.0	167	190	173
14	1.3	229	1.5	115	19.9	194	1.8	138	562	225
15	1.4	198	1.9	146	23.5	184	2.8	187	456	182
16	0.6	132	0.9	113	12.9	158	1.5	167	311	194
17	1.5	264	1.8	138	32.6	317	2.9	223	5645	225
18	1.0	189	1.5	136	15	157	1.7	142	311	148
19	1.0	142	1.1	85	19.8	155	1.8	120	297	119
20	1.2	169	1.7	121	27.9	217	2.7	180	419	167

^1^ For additional data including vitamin intake standards, see Table S3. Concerns also Table 8.

**Table 8:** Dietary intake of selected vitamins in subjects with PWS–part 2.

	Vitamin B_12_		Vitamin C		Vitamin A (Retinol Equivalent)		Vitamin D		Vitamin E	
No.	Average Intake (µg)	EAR Coverage (%)	Average Intake (mg)	EAR Coverage (%)	Average Intake (µg)	EAR Coverage (%)	Average Intake (µg)	Assessment of the Implementation	Average Intake (mg)	Assessment of the implementation
1	3.9	98	213	237	1134	199	1.3	insufficient	8.1	insufficient
2	2.3	58	58.9	65	621	109	1	insufficient	5.8	insufficient
3	2.3	58	89.3	119	656	134	1.8	insufficient	4.8	insufficient
4	6.2	177	91.8	153	857	179	4.6	insufficient	7.0	insufficient
5	3.7	106	76.3	127	1207	251	7.3	insufficient	8.9	insufficient
6	4.2	120	123	205	1778	371	5.8	insufficient	16.2	adequate
7	2.7	77	148	247	1232	257	2.8	insufficient	7.5	insufficient
8	2	133	226	904	1197	488	1.3	insufficient	7.8	insufficient
9	2.6	104	97.1	243	1244	389	6	insufficient	7.5	insufficient
10	5.8	145	153	170	976	171	2.6	insufficient	8.0	insufficient
11	3.2	213	3.2	21	1473	719	3	insufficient	10.4	adequate
12	1.6	40	146	162	1209	212	1.2	insufficient	8.6	insufficient
13	0.9	60	105	419	6846	279	1.5	insufficient	4.7	insufficient
14	1.5	38	2887	360	1229	251	1.8	insufficient	9.2	insufficient
15	3.8	95	231	257	1462	256	1.7	insufficient	10.6	insufficient
16	2.6	104	188	471	1116	349	2.4	insufficient	7.1	insufficient
17	5.8	145	388	485	1986	405	7	insufficient	16.7	adequate
18	2.2	63	157	262	1311	273	1	insufficient	6.9	insufficient
19	1.9	48	138	153	937	164	0.8	insufficient	6.2	insufficient
20	5.1	128	231	271	1459	252	3.7	insufficient	9.6	insufficient

**Table 9:** Comparison of vitamin intake across sex and age groups.

	All Subjects	All Males	All Females	Males 2–17 Years Old	Females 2–17 Years Old	Males 18–28 Years Old	Females 18–28 Years Old
Vitamin B_1_ (mg)	1.09 ± 0.25	1.04 ± 0.25	1.15 ± 0.25	1.00 ± 0.23	1.07 ± 0.23	1.08 ± 0.29	1.4 ± 0.14
Vitamin B_2_ (mg)	1.43 ± 0.45	1.43 ± 0.44	1.43 ± 0.50	1.30 ± 0.43	1.35 ± 0.56	1.55 ± 0.45	1.65 ± 0.21
Vitamin B_3_ (mg)	17.1 ± 5.9	17.9 ± 4.8	15.9 ± 7.4	17.4 ± 5.5	12.5 ± 1.9	18.5 ± 4.5	26.3 ± 9.0
Vitamin B_6_ (mg)	1.82 ± 0.55	1.87 ± 0.57	1.74 ± 0.53	1.82 ± 0.47	1.53 ± 0.27	1.92 ± 0.70	2.35 ± 0.78
Folic acid (µg)	343 ± 99.0	327 ± 68.7	368 ± 134	316 ± 54.7	303 ± 70.9	338 ± 84.3	563 ± 1.06
Vitamin B_12_ (µg)	3.22 ± 1.55	3.52 ± 1.52	2.76 ± 1.59	3.82 ± 1.52	2.47 ± 1.12	3.22 ± 1.59	3.65 ± 3.04
Vitamin C (mg)	158 ± 88.0	148 ± 59.2	172 ± 123	139 ± 61.35	117 ± 74.0	157 ± 61.3	338 ± 71.06
Vitamin A (retinol equivalent) (µg)	1188 ± 350	1121 ± 243	1289 ± 469	1186 ± 197	1183 ± 443	1056 ± 284	1608 ± 535
Vitamin D (µg)	2.93 ± 2.10	2.95 ± 2.08	2.40 ± 1.80	4.47 ± 1.90	2.4 ± 1.80	1.43 ± 0.65	4.4 ± 3.68
Vitamin E (mg)	8.58 ± 3.13	7.91 ± 1.37	8.47 ± 4.34	7.93 ± 1.06	8.47 ± 4.34	7.88 ± 1.74	12.95 ± 5.30

**Table 10:** Comparison of mineral intake across sex and age groups.

	All Subjects	All Males	All Females	Males 2–17 Years Old	Females 2–17 Years Old	Males 18–28 Years Old	Females 18–28 Years Old
Sodium (mg)	1591 ± 450	1654 ± 527	1496 ± 309	1866 ± 557	1407 ± 292	1441 ± 440	1764 ± 226
Potassium (mg)	3229 ± 7135	3321 ± 747	3092 ± 683	3172 ± 577	2816 ± 489	3469 ± 918	3921 ± 491
Calcium (mg)	824 ± 249	854 ± 234	779 ± 280	866 ± 211	775 ± 321	841 ± 274	791 ± 180
Phosphorus (mg)	1290 ± 302	1324 ± 312	1240 ± 300	1370 ± 312	1182 ± 263	1277 ± 334	1414 ± 45
Magnesium (mg)	331 ± 72.9	322 ± 73.4	343 ± 75.3	313 ± 54.3	314 ± 59.0	332 ± 93.3	429 ± 50.8
Iron (mg)	11.4 ± 3.08	10.9 ± 2.03	12.1 ± 4.27	10.8 ± 2.33	11.1 ± 4.46	11.0 ± 1.92	15.3 ± 1.13
Zinc (mg)	8.90 ± 1.95	9.11 ± 2.14	8.59 ± 1.70	9.02 ± 2.25	7.93 ± 1.28	9.20 ± 2.34	10.6 ± 1.34
Copper (mg)	1.14 ± 0.29	1.09 ± 0.26	1.20 ± 0.35	1.05 ± 0.27	1.07 ± 0.28	1.13 ± 0.27	1.6 ± 0.14
Iodine (mg)	37.4 ± 15.1	41.9 ± 12.7	30.7 ± 16.6	43.6 ± 12.4	26.0 ± 14.5	40.3 ± 14.1	45.0 ± 18.5
Manganese (mg)	5.24 ± 1.59	5.23 ± 1.60	5.25 ± 1.67	5.35 ± 1.54	4.78 ± 1.68	5.10 ± 1.80	6.65 ± 0.35

**Table 11:** Dietary intake of selected minerals in subjects with PWS–part 1 ^1^.

	Sodium		Potassium		Calcium		Phosphorus		Magnesium	
No.	Average Intake (mg)	Assessment of the Implementation	Average Intake (mg)	Assessment of the Implementation	Average Intake (mg)	EAR Coverage (%)	Average Intake (mg)	Assessment of the Implementation	Average Intake (mg)	Assessment of the Implementation
1	17,042	insufficient	4397	adequate	1316	153	1773	adequate	424	adequate
2	889	insufficient	2223	insufficient	690	80	887	adequate	230	adequate
3	1416	insufficient	2777	insufficient	538	63	1194	adequate	259	adequate
4	2196	adequate	3262	adequate	1169	122	1660	adequate	340	adequate
5	1961	insufficient	3075	adequate	918	96	1532	adequate	306	adequate
6	1648	insufficient	2722	adequate	1157	120	13,917	adequate	417	adequate
7	1379	insufficient	3710	adequate	941	98	1322	adequate	313	adequate
8	998	insufficient	2870	adequate	685	101	925	adequate	265	adequate
9	1503	insufficient	2480	adequate	760	112	1178	adequate	292	insufficient
10	1293	insufficient	3463	insufficient	845	98	1477	adequate	368	adequate
11	1682	adequate	3493	adequate	1151	295	1477	adequate	343	adequate
12	1006	insufficient	3386	insufficient	751	100	1065	adequate	310	insufficient
13	1107	insufficient	1995	adequate	373	55	814	adequate	292	adequate
14	1605	insufficient	3574	adequate	664	77	1093	adequate	393	adequate
15	1843	insufficient	4595	adequate	935	109	1418	adequate	436	adequate
16	1380	insufficient	2596	adequate	533	78	868	adequate	231	adequate
17	1924	insufficient	4269	adequate	919	107	1734	adequate	465	adequate
18	1592	insufficient	3037	adequate	746	78	1294	adequate	311	adequate
19	1911	insufficient	2749	insufficient	509	68	1042	adequate	222	insufficient
20	2776	adequate	3910	adequate	877	91	1663	adequate	396	adequate

^1^ For additional data including mineral intake standards see Table S4. Concerns also Table 12.

**Table 12:** Dietary intake of selected minerals in subjects with PWS–part 2.

	Iron		Zinc		Copper		Iodine		Manganese	
No.	Average Intake (mg)	EAR Coverage (%)	Average Intake (mg)	EAR Coverage (%)	Average Intake (mg)	Assessment of the Implementation	Average Intake (µg)	Assessment of the Implementation	Average Intake (mg)	Assessment of the Implementation
1	12.6	210	11.3	122	1.4	insufficient	52.5	insufficient	5.1	adequate
2	8.6	143	5.7	61	0.8	insufficient	21.6	insufficient	2.4	insufficient
3	9.0	129	8.0	81	1.1	adequate	17.1	insufficient	4.3	adequate
4	10.4	130	10.9	122	1.1	adequate	54.2	insufficient	5.1	adequate
5	10.7	134	9.2	103	1.1	insufficient	36.7	insufficient	4.6	adequate
6	20.0	250	9.0	101	1.6	adequate	22.1	insufficient	8.2	adequate
7	12.3	154	8.4	94	1.2	insufficient	31.8	insufficient	3.9	adequate
8	8.6	172	6.8	148	1.0	adequate	20.5	insufficient	4.0	adequate
9	9.2	115	7.2	116	0.9	insufficient	39.3	insufficient	7.0	adequate
10	11.8	197	10.0	108	1.3	insufficient	38.0	insufficient	5.9	adequate
11	10.5	210	9.2	256	1.0	adequate	54.0	insufficient	4.0	adequate
12	9.6	160	7.9	85	1.0	insufficient	44.8	insufficient	4.8	adequate
13	8.2	164	6.0	130	0.9	insufficient	27.7	insufficient	3.9	adequate
14	14.5	207	9.6	126	1.5	adequate	31.9	insufficient	6.9	adequate
15	13.4	223	11.6	125	1.4	insufficient	57.4	insufficient	7.9	adequate
16	7.9	99	6.2	100	0.6	insufficient	36.0	insufficient	4.0	adequate
17	16.1	230	11.5	151	1.7	adequate	58.1	insufficient	6.4	adequate
18	10.1	144	8.6	97	0.8	insufficient	14.3	insufficient	4.3	adequate
19	9.7	162	8.7	94	0.9	insufficient	27.2	insufficient	4.5	adequate
20	14.5	181	12.2	103	1.4	adequate	63.3	insufficient	7.5	adequate

## Data Availability

The dietary records of researched PWS persons are available on request (in Polish).

## Supplementary Materials

The following supporting information can be downloaded at: https://www.mdpi.com/article/10.3390/nu15173811/s1, Table S1. Nutritional status in the studied group–supplementary data. Table S2. Recommended intake of energy and macronutrients in subjects with PWS–supplementary data. Table S3. Recommended intake of selected vitamins in subjects with PWS–supplementary data. Table S4. Recommended intake of selected minerals in subjects with PWS–supplementary data.
