# Supplementary material for: Analysis of the Diet Quality and Nutritional State of Children, Youth, and Young Adults with Prader–Willi Syndrome: A Polish Multiple Case Study
Source: Nutrients. 2023 Aug 31;15(17):3811. doi: 10.3390/nu15173811 (PMC10490043; doi:10.3390/nu15173811)
Supplement: Supplementary file 1 [file nutrients-15-03811-s001.zip › nutrients-2532288-supplementary.pdf]

Table S1. Nutritional status in the studied group – supplementary data

No.	Body mass [kg]	Height [cm]	BMI [kg/m²]	PAL
1.	68.0	175.0	22.2	1.6
2.	53.6	175.2	17.4	1.6
3.	69.1	162.2	26.4	1.4
4.	46.2	156.0	19.0	1.6
5.	35.5	152.0	15.4	1.6
6.	39.3	147.8	18.2	1.75
7.	32.0	135.0	17.6	1.6
8.	12.3	90.4	16.9	1.6
9.	28.2	137.5	14.9	1.6
10.	76.0	182.0	23.0	1.6
11.	11.7	86.0	15.8	1.75
12.	53.0	160.0	20.7	1.75
13.	17.9	109.0	15.0	1.75
14.	69.9	153.0	29.9	1.4
15.	77.0	173.0	25.8	1.6
16.	21.5	119.0	15.1	1.75
17.	104.2	167.0	37.3	1.4
18.	50.0	160.0	19.5	1.6
19.	128.0	165.0	47.0	1.4
20.	40.7	163.5	15.2	1.6

Table S2. Recommended intake of energy and macronutrients in subjects with PWS – supplementary data

No.	Protein 10-20% EER [g]	Fat 20-35% EER [g]	SFA 6% EER [g]	Carbohydrates 45 – 65 % EER [g]	Fiber AI [g]
1	49.7 - 99.4	44.2 – 77.3	13,3	224 - 323	25
2	63.8 – 127.5	56.7 – 99.2	17,0	287 – 414	25
3	39.4 – 78.8	35.0 – 61.3	10,5	177 – 256	21
4	56.3 – 112.5	50.0 – 87.5	15,0	253 – 366	19
**5**	44.1 – 88.2	39.2 – 68.6	11,8	198 – 287	19
**6**	46.0 – 91.9	40.8 – 71.5	12,3	207 – 299	19
**7**	44.1 – 88.2	39.2 – 68.6	11,8	198 – 287	19
**8**	26.3 – 52.5	23.3 – 40.8	7,0	118 – 171	14
**9**	44.1 – 88.2	39.2 – 68.6	11,8	198 – 287	16
**10**	54.4 – 108.8	48.3 – 84.6	14,5	245 – 353	25
**11**	18.8 – 37.5	16.7 – 29.2	5,0	84 – 122	10
**12**	30.6-61.3	27.2 – 47.6	8,2	138 – 199	25
**13**	26.3 – 52.5	23.3 – 40.8	7,0	118 – 171	14
**14**	33.8 – 67.5	30.0 – 52.5	9,0	152 – 219	25
**15**	49.7 – 99.4	44.2 – 77.3	13,3	224 – 323	25
**16**	39.4 – 78.8	35.0 – 61.3	10,5	177 – 256	16
**17**	25.6 – 51.3	22.8 – 39.9	6,8	115 – 167	25
**18**	39.4 – 78.8	35.0 – 61.3	10,5	177 – 256	19
**19**	26.3 – 52.5	23.3 – 40.8	7,0	118 – 171	25
**20**	56.3 – 112.5	50,0 – 87.5	15,0	253 - 366	21

Table S3. Recommended intake of selected vitamins in subjects with PWS – supplementary data

No.	Vitamin B_1 MJ	Vitamin B_1 EAR [mg]	Vitamin B_2 EAR [mg]	Vitamin B_3 EAR [mg]	Vitamin B_6 EAR [mg]	Folic acid EAR [µg]	Vitamin B_{12} EAR [µg]	Vitamin C EAR [mg]	Vitamin A (retinol equivalent) EAR [µg]	Vitamin D AI [µg]	Vitamin E AI [mg]
1	9.8	0.71	1.3	12.7	1.5	250	4.0	90	570	15	13
2	9.8	0.71	1.3	12.7	1.5	250	4.0	90	570	15	13
3	9.3	0.67	1.4	12.1	1.3	250	4.0	75	490	15	11
4	8.6	0.62	1.1	11.2	1.2	210	3.5	60	480	15	13
5	7.4	0.53	1.1	9.6	1.2	210	3.5	60	480	15	13
6	7.0	0.50	1.1	9.1	1.2	210	3.5	60	480	15	11
7	7.4	0.53	1.1	9.6	1.2	210	3.5	60	480	15	13
8	4.9	0.35	0.6	6.4	0.6	110	1.5	25	245	15	9
9	7.1	0.51	0.8	9.2	0.9	160	2.5	40	320	15	13
10	9.8	0.71	1.3	12.7	1.5	250	4.0	90	570	15	13
11	4.6	0.33	0.5	6.0	0.5	90	1.5	15	205	15	6
12	9.8	0.71	1.3	12.7	1.5	250	4.0	90	570	15	13
13	5.9	0.42	0.6	7.7	0.6	110	1.5	25	245	15	9
14	7.9	0.57	1.3	10.3	1.3	250	4.0	80	490	15	11
15	9.8	0.71	1.3	12.7	1.5	250	4.0	90	570	15	13
16	6.3	0.45	0.8	8.2	0.9	160	2.5	40	320	15	9
17	7.9	0.57	1.3	10.3	1.3	250	4.0	80	490	15	11
18	7.6	0.53	1.1	9.6	1.2	210	3.5	60	480	15	11
19	9.8	0.71	1.3	12.7	1.5	250	4.0	90	570	15	13
20	9.9	0.71	1.4	12.9	1.5	250	4.0	85	580	15	13

Table S4. Recommended intake of selected minerals in subjects with PWS – supplementary data

No.	Sodium AI [mg]	Potassium AI [mg]	Calcium EAR [mg]	Phosphorus AI [mg]	Magnesium AI [mg]	Iron EAR [mg]	Zinc EAR [mg]	Copper AI [mg]	Iodine AI [mg]
1	2000	3500	860	550	350	6.0	9.3	1.6	150
2	2000	3500	860	550	350	6.0	9.3	1.6	150
3	2000	3500	860	640	250	7.0	9.9	1.1	130
4	2000	2700	960	640	300	8.0	8.9	1.3	120
5	2000	2700	960	640	300	8.0	8.9	1.3	120
6	2000	2700	960	640	250	8.0	8.9	1.1	120
7	2000	2700	960	640	300	8.0	8.9	1.3	120
8	1300	1100	680	440	230	5.0	4.6	1.0	90
9	1700	1800	680	440	300	8.0	6.2	1.3	90
10	2000	3500	860	550	350	6.0	9.3	1.6	150
11	1100	800	390	250	170	5.0	3.6	0.7	90
12	2000	3500	750	550	350	6.0	9.3	1.6	150
13	1300	1100	680	440	230	5.0	4.6	1.0	90
14	2000	3500	860	550	300	7.0	7.6	1.3	150
15	2000	3500	860	550	350	6.0	9.3	1.6	150
16	1700	1800	680	440	230	8.0	6.2	1.0	90
17	2000	3500	860	550	300	7.0	7.6	1.3	150
18	2000	2700	960	640	250	7.0	8.9	1.1	120
19	2000	3500	750	550	350	6.0	9.3	1.6	150
20	2000	3500	960	640	300	8.0	11.8	1.3	130
